# Cardiogenic Shock due to Psychosis-Induced Inverted Takotsubo Cardiomyopathy Bridged-to-Recovery with a Percutaneous Left Ventricular Assist Device

**DOI:** 10.1155/2016/5498650

**Published:** 2016-12-12

**Authors:** Ravi Korabathina, Warren Abel, Arthur Labovitz

**Affiliations:** ^1^Department of Cardiovascular Sciences, University of South Florida Morsani College of Medicine/Bayfront Health Medical Center, 625 Sixth Avenue South, Suite 430 St. Petersburg, FL 33703, USA; ^2^Department of Pulmonary and Critical Care, Bayfront Health Medical Center, St. Petersburg, FL, USA; ^3^Department of Cardiovascular Sciences, University of South Florida Morsani College of Medicine, Tampa, FL, USA

## Abstract

Inverted Takotsubo cardiomyopathy, a less common variant in the spectrum of stress-induced cardiomyopathy, is increasingly being reported. This report describes an acute psychiatric illness leading to the onset of this syndrome. The patient presented here developed cardiogenic shock but successfully recovered with the use of a percutaneous left ventricular assist device.

## 1. Introduction

Stress-induced cardiomyopathy (SIC) has been characterized as a syndrome of transient left ventricular (LV) systolic dysfunction that occurs in the absence of obstructive coronary artery disease and that is provoked by physical or emotional distress. The less common SIC variant of inverted Takotsubo cardiomyopathy (ITC) with its unique contractile pattern of basal and mid-LV wall akinesis but normal apical movement is increasingly becoming recognized. While its clinical presentation is typically nonthreatening, requiring only supportive care, there are rare instances of ITC presenting catastrophically with cardiogenic shock. Such a unique case and its successful treatment are described herein.

## 2. Case Presentation

A 51-year-old female, who is originally from Italy, presented to the Emergency Room (ER) 24 hours after landing in the United States with increasing psychosis. Her history was only remarkable for paranoid schizophrenia, but she was highly functional at baseline. As per her family, she was hearing command hallucinations which did not improve with her usual antipsychotic medications, aripiprazole and haloperidol. Her haloperidol dosing was actually being tapered over the previous 4 months. She had no history of drug or alcohol use.

**Figure 1:**
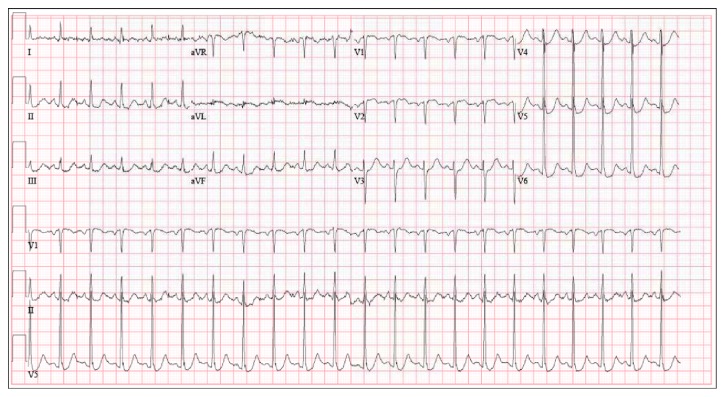
Electrocardiogram at admission showing sinus tachycardia and diffuse ST segment depressions.

**Figure 2:**
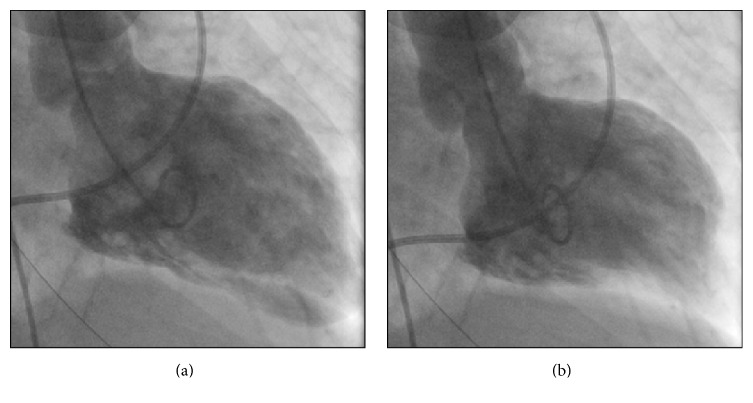
(a) Left ventriculogram cine capture in right anterior oblique 30 degrees' projection at end-diastole. (b) Left ventriculogram cine capture at end-systole showing apical contraction only with akinesis of the base and mid wall segments.

In addition to increasing psychomotor agitation, her only other complaints were profound weakness and dyspnea. Her initial vital signs revealed a systolic blood pressure of 70 mmHg, a heart rate of 118 beats per minute, and oxygen saturations via pulse oximetry of 78% on room air. The chest X-ray revealed bilateral infiltrates concerning for congestive heart failure. She ultimately progressed to requiring mechanical ventilatory support and within the first few hours in the ER was necessitating escalating doses of 3 vasopressors. Her electrocardiogram showed precordial ST segment depressions (Figure 1), and the laboratory parameters were remarkable for an elevated troponin I of 14.7 ng/mL and elevated plasma brain natriuretic peptide of 1760 pg/mL. With a clinical diagnosis of worsening cardiogenic shock, she was taken urgently to the cardiac catheterization laboratory. The coronary arteries were noted to be patent, but left ventriculography revealed severe systolic dysfunction with an ejection fraction approximated at 15–20%. The ventricular wall segment analysis revealed hyperdynamic apical wall contractility and akinesis of the basal and mid ventricular walls (Figure 2), a pattern consistent with an ITC. The LV end-diastolic pressure was elevated at 23 mmHg, and pulmonary artery catheter indices showed a low cardiac output of 1.7 L/min. Immediate echocardiography confirmed normal right ventricular size and systolic function, and there was no evidence of significant valvular disease or pericardial effusion.

**Figure 3:**
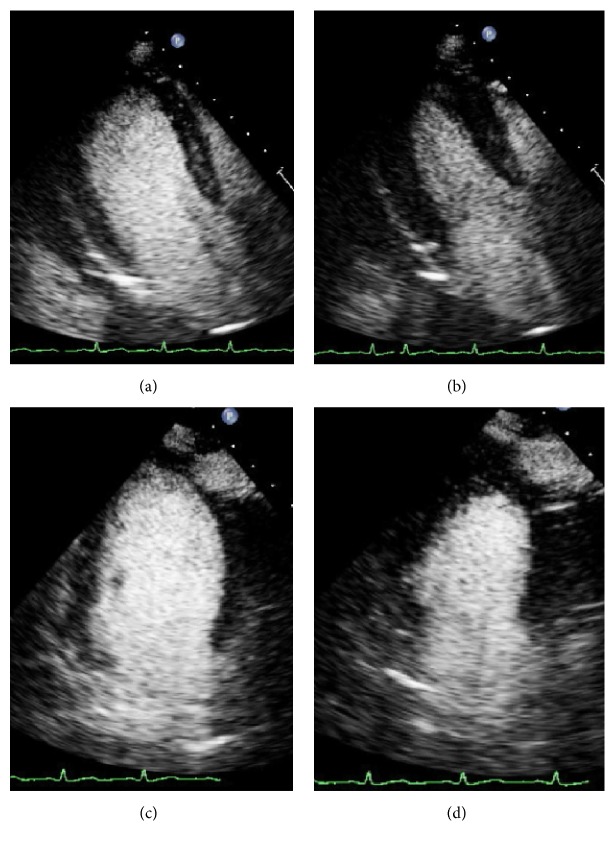
(a) Contrast transthoracic echocardiogram 3-chamber view of left ventricular cavity at end-diastole. (b) 3-chamber view at end-systole showing global contraction. (c, d) 4-chamber view of left ventricular cavity at end-diastole and end-systole, respectively.

The decision was made to proceed with left ventricular assist device (LVAD) support using an Impella 2.5 LP (Abiomed Inc., Danvers, MA) device. Over the ensuing 24 hours following Impella LVAD placement, phenylephrine, norepinephrine, and vasopressin were all tapered off. Her hemodynamic indices showed steady improvement (Table 1), and the Impella LVAD was removed 60 hours following initial placement. Her native cardiac output 24 hours following device explantation had increased to 5.4 L/min. She was weaned from mechanical ventilatory support and extubated on her fifth day of hospitalization. On the same day, a contrast echocardiography showed normalization in LV systolic function with an ejection fraction of 55–60% (Figure 3). The previous akinesis involving the mid and basal ventricular wall segments was no longer present. A repeat chest X-ray showed significant clearing of the previously seen vascular congestion. She was started on a low dose of carvedilol and lisinopril and, after modification of psychotropic medications, was discharged on the seventh day of hospitalization without any evidence of psychosis and with near full baseline functionality. One year later, an email reply from the patient's primary doctor in Italy communicated that she was still doing well with a follow-up echocardiogram showing stable cardiac function.

## 3. Discussion

Inverted Takotsubo cardiomyopathy has been described as one of the 4 patterns of SIC, with the most predominant being the classical Takotsubo cardiomyopathy (CTC) with its characteristic LV apical-ballooning. With ITC, the pattern of contractile dysfunction is different from CTC and localized to the basal and mid-LV wall segments but the apical wall shows preserved contractility. Two case series, totaling 163 patients, have compared clinical features of ITC to CTC showing the former cohort as being comprised of younger individuals and universally having an identifiable stress trigger [1, 2]. Interestingly, the ITC subjects in these two series presented infrequently with cardiogenic shock when compared to CTC subjects.

We present here an original case of ITC complicated by cardiogenic shock that was precipitated by worsening psychosis in an individual with schizophrenia who was suboptimally treated with antipsychotic medications. While the patient herein fell within the age group and gender predilection expected for the typical ITC subject, she also demonstrated unusual features. For one, mental illness exacerbation, as an emotional source of stress, is a unique addition to the list of primarily physical stressors that have been described to bring forth ITC, such as neurologic insult and post-surgical/procedural state [3, 4]. Emotional triggers arising from mental illness have been described to precipitate the more classic forms of SIC [5]. Secondly, ITC typically presents with stable hemodynamics and seldom with shock. In fact, there have only been 4 documented ITC cases in the literature demonstrating refractory cardiogenic shock necessitating the use of percutaneous mechanical support devices, and the triggers were all physical stressors [6–9]. An intra-aortic balloon pump was needed to stabilize two cases of ITC shock related to encephalitis and a dental procedure, and percutaneous extracorporeal membrane oxygenation was required for two other cases of ITC shock related to pheochromocytoma and iatrogenic epinephrine injection. The factors that led to hemodynamic instability in this limited number of ITC cases are unclear. While physical versus emotional stressors have been purported to confer added risk [10], the current case demonstrates the serious impact of even an emotional stressor, such as mental illness exacerbation, in bringing forth cardiogenic shock.

While the specific pattern of SIC does not appear to carry prognostic implications, the in-hospital mortality for SIC is high at 8.7% based upon the largest case series of 208 patients [10]. In multivariate analysis, shock presentation carried a 3.7 times higher odds of mortality. The leading hypothesis regarding the pathophysiology of SIC is that a catecholamine surge occurs after a stressful event, and targets beta-adrenergic receptors resulting in a negative inotropic effect. There is variation in the density and distribution of these adrenoreceptors along the ventricular myocardium based upon age, and this accounts for the localized areas of myocardial stunning [11]. This explains the predominant occurrence of the ITC pattern in younger patients, as the density of adrenoreceptors is greatest at the basal and mid-LV wall segments in younger age groups. In the context of ITC shock, the use of certain vasoactive agents with their beta-receptor agonist properties is paradoxical and seemingly may worsen the hemodynamic condition. While there is limited evidence regarding the treatment approach to these critically ill patients, the earlier incorporation of mechanical LVADs into the treatment algorithm of ITC shock patients appears logical. To our knowledge, the efficacious and safe use of the percutaneous Impella continuous axial flow LVAD to bridge a critically ill ITC shock patient rapidly to complete recovery has not been previously reported.

## Figures and Tables

**Table 1:** Hemodynamic indices and laboratory values during various stages of heart failure treatment.

Hemodynamic indices	Before Impella	During Impella (24 hours)	After Impella removal (24 hours)
Mean arterial pressure (mmHg)	72	84	75
Systolic blood pressure (mmHg)	82	104	114
Diastolic blood pressure (mmHg)	66	74	56
Heart rate (beats/min)	112	97	98
Right atrial pressure (mmHg)	12	10	4
Pulmonary artery pressure mean (mmHg)	22	24	17
Pulmonary capillary wedge pressure (mmHg)	18	20	11
Cardiac index (L/min/m^2^)	1.05	2.10	3.20
Cardiac output (L/min)	1.70	3.50	5.40
Impella flow (L/min)	0	2.20	0
Systemic vascular resistance (dyne/sec/cm^2^)	2824	1691	1051
Pulmonary artery oxygen saturation, %	36	72	74
Femoral artery oxygen saturation, %	100	100	100

Laboratory values	Before Impella	During Impella (24 hours)	After Impella removal (24 hours)

Sodium, mEQ/L	137	135	138
Blood urea nitrogen, mg/dL	17	9	8
Creatinine, mg/dL	1.30	0.70	0.70
CO_2_, mmol/L	20.8	26.4	22.9
Alanine transaminase, IU/L	30	766	219
Aspartate transaminase, IU/L	84	379	41
Hemoglobin, g/dL	14.6	10.5	10.9
Platelets, K/mL	307	116	174
White blood cell, /microL	16.0	12.3	9.6
Arterial pH	7.26	7.54	7.44
Lactate, IU/L	6.2	1.6	0.7
Troponin I, ng/mL	14.7	13.1	
Brain natriuretic peptide, pg/mL	1760		256
